# Leaf Developmental Stages Strongly Modulate Indole Emissions in Response to Simulated Insect Herbivory

**DOI:** 10.3390/plants14243761

**Published:** 2025-12-10

**Authors:** Marie Engelberth, Jurgen Engelberth

**Affiliations:** Department of Biology, Health, and the Environment, The University of Texas at San Antonio, San Antonio, TX 78249, USA; marie.engelberth@utsa.edu

**Keywords:** indole, herbivory, herbivore induced plant volatiles, insect elicitor, plant defense, gene expression, leaf development, *Zea mays*

## Abstract

Maize plants challenged by insect herbivores activate an array of defense measures, all aimed to reduce damage and repel the attacker . Among those are the activation of proteins that interfere with the digestion of consumed plant material in the herbivore (proteinase inhibitors), the production of toxic compounds like benzoxazinoids, and the biosynthesis and emission of herbivore-induced plant volatiles (HIPVs). Among those HIPVs are mainly a variety of terpenoids, green leaf volatiles (GLVs), and indole. While often serving as attractants for natural enemies of the attacking herbivores, many of those volatiles have also been found to induce defense responses in neighboring plants and/or prime them against future menace. Indole is of particular interest since it can be involved in a variety of biosynthetic pathways including those leading to auxin, benzoxazinoids, and tryptophan. Here, we demonstrate that indole emissions in response to simulated insect herbivory by treatment with an insect elicitor (N-linolenoyl glutamine) strongly depend on the developmental status of the affected leaf in maize. Outgrown leaves emit significantly higher amounts of indole compared to the next younger, still growing leaves, distinguishing indole from other HIPVs, which are typically released at higher levels by young leaves. As a central and flexible metabolic intermediate, indole emissions appear to be mediated through variable allocation between growth-related processes and defense-associated outcomes, depending on the developmental stage of the damaged leaf. These findings highlight the importance of considering plants as inherently dynamic organisms.

## 1. Introduction

Plants are constantly attacked by herbivores and pathogens. To survive in this generally hostile environment, plants have developed an array of defensive strategies. Aside from constitutive defenses, which include permanent physical and chemical traits, induced defenses are temporary protective mechanisms that are activated in response to a herbivore.

Among those inducible measures is the activation of chemical defenses, which may directly and/or indirectly affect pests and pathogens. In particular, indirect defenses in the form of volatiles produced by plants upon herbivory have come into focus due to their function in tritrophic interactions between plants, herbivores, and their natural enemies [1,2]. These herbivore-induced plant volatiles (HIPVs) have also been found to be sensed by other plants nearby, where they can induce defensive measures, resulting in better preparedness should herbivores move on [3,4,5]. Among those HIPVs that can induce defensive measures and have been well characterized are green leaf volatiles (GLVs) and indole [4,5]. Both were found to mainly induce a primed response, meaning that plants that received these signals start preparing their defenses, leading to better protection. However, while GLVs are emitted immediately after being damaged, indole is usually produced upon insect herbivory at a much later time point, often several hours after the initial elicitation [5]. And while the biosynthesis of GLVs is rather straightforward, with mainly volatile 6-carbon aldehydes, alcohols, and esters being produced [6], indole biosynthesis is much more complex, with several biosynthetic pathways producing and utilizing this compound as an intermediate or end product [7,8,9].

Indole (2,3-Benzopyrrole), for example, is an important intermediate in tryptophane biosynthesis, and since tryptophane is a precursor of indole acetic acid (IAA), the major auxin in plants, indole plays a crucial role in the production of this essential plant hormone [10]. Although alternative biosynthetic pathways for IAA biosynthesis exist, all of them require indole as a key intermediate [7]. Further, indole is also a precursor for benzoxazinoid biosynthesis. Benzoxazinoids are important plant defense compounds, in particular in maize. They are rapidly produced in response to herbivory and pathogen infections and are quite effective in reducing damage by these threats [7,8].

In contrast to the biosynthetic pathways described above, indole can also be the end product of its own pathways and as such is often part of the HIPVs. A key enzyme in this process is indole-3-glycerol phosphate lyase (*IgL*), which releases indole from its precursor, indole-3-glycerol phosphate [7,8,9]. The expression of *IgL* is strongly induced by insect-derived elicitors such as volicitin, a conjugate of a 17-hydroxylated linolenic acid with glutamine [11]. Once emitted, indole has the ability to prime defenses in other plants nearby, resulting in an enhanced production of jasmonates and an increased release of HIPVs [5,12,13]. Aside from priming jasmonate accumulation, indole also appears to act through a mitogen-activated protein kinase pathway (MAPK) [12,13], which regulates at least some of the defense responses in maize [14]. However, indole does not activate MAPK directly upon exposure, but rather primes this signaling pathway, thereby enhancing its signaling strength during insect herbivory [13].

Due to its multifaceted roles in multiple pathways and defense responses, it is not surprising that the biosynthesis and activity of indole is highly context-dependent. Environmental factors as well as the type of biotic stressor may impact the biosynthesis and thus the defensive efficiency of indole in nature. Here, we present data demonstrating the influence of different developmental stages on the insect elicitor-induced indole emissions and *IgL* transcript accumulation in maize, using N-linolenoyl glutamine as our elicitor [15]. We compare those results with expression data on other defense-related genes like cysteine protease inhibitor (*CPI*) and terpene synthase 10 (*TPS 10*). Our findings provide strong evidence that indole fulfills functions beyond direct defense and therefore differs substantially from other compounds inducing defensive responses in the plant.

## 2. Results

### 2.1. Volatile Emissions After Insect Elicitor Treatment

**Figure 1:**
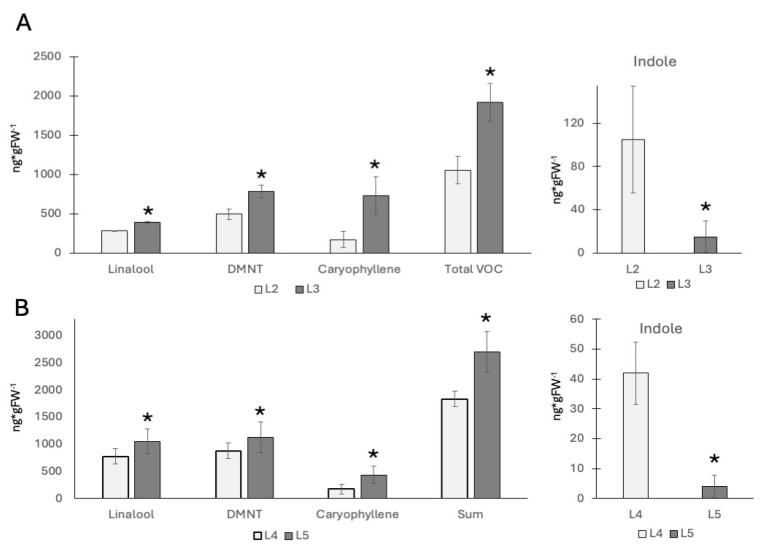
Volatile emissions from maize seedlings in response to insect elicitor treatment (IE). N-linolenoyl-glutamine was used as an elicitor. (**A**) V2 stage plants. The second and third leaves were treated with IE. The left panel shows major terpenes. The right panel shows indole emissions. (**B**) V4 stage plants. The fourth and fifth leaves were treated with IE. The left panel shows major terpenes. The right panel shows indole emissions. * indicates significant differences (*t* test; *p* ≤ 0.05; *N* = 3).

Previous reports on volatile emissions from maize seedlings have shown that younger leaves emit more volatiles when compared to older leaves of the same plant [16]. And while those studies used GLVs to elicit volatile production, we expected to find the same for insect elicitor-induced volatiles. To confirm this hypothesis, we used V2 and V4 plants and treated them with a pure insect elicitor (IE; here: N-linolenoyl glutamine) as described previously [15]. Since IEs induce HIPVs and most other defenses mainly in the region upwards (distal) from the treatment site [17,18], we focused our studies on this portion of the treated leaves. For volatile terpenes including linalool, DMNT, and caryophyllene, we confirmed that younger leaves produce/release significantly more of these HIPVs (Figure 1A). In V2 maize plants, we found 284 ± 4 ng/g fresh weight (FW) for linalool, 494 ± 70 ng/g FW for 4,8-dimethyl-1,3,7-nonatriene (DMNT), and 171 ± 101 ng/g FW for caryophyllene in the second leaf, and the third leaf produced 388 ± 12 ng/g FW for linalool, 782 ± 78 ng/g FW for DMNT, and 731 ± 238 ng/g FW for caryophyllene. However, significant differences between the second (mature) and the third (growing) leaf were found for all individual HIPV compounds as well as the summarized HIPVs (Figure 1A). In contrast, indole emissions were significantly higher in the second leaf (104 ± 49 ng/g FW) than in the third leaf (19 ± 14 ng/g FW). No significant amounts of HIPVs were found in control plants.

For the older V4 plants, we found similar results for the fourth (mature) and fifth (growing) leaf (Figure 1B). Here, we found 777 ± 225 ng/g FW for linalool, 879 ± 286 ng/g FW for DMNT, and 172 ± 161 ng/g FW for caryophyllene in the fourth leaf, and 1051 ± 140 ng/g FW for linalool, 1127 ± 286 ng/g FW for DMNT, and 430 ± 161 ng/g FW for caryophyllene in the fifth leaf. Compared to the younger V2 stage seedling, indole levels followed the opposite trend. Here, 42 ± 10 ng/g FW were found in the fourth leaf, while only 4 ± 3 ng/g FW were detected in the growing fifth leaf. Again, no significant amounts of HIPV were found in control plants.

These results confirm a generally higher production of HIPVs from younger leaves, as shown in a previous study [15]. However, the indole emissions do not follow this trend. Volatile indole emission after insect elicitor treatment was always much lower in younger, still growing leaves. This may suggest that younger growing leaves appropriate resources differently from older and outgrown leaves. To further investigate, we performed gene expression studies related to indole biosynthesis (indole-3-glycerol lyase (IgL)), terpene biosynthesis (terpene synthase 10 (TPS 10)), and direct defense (cysteine protease inhibitor (CPI)).

### 2.2. Defense Gene Expression After Insect Elicitor Treatment

**Figure 2:**
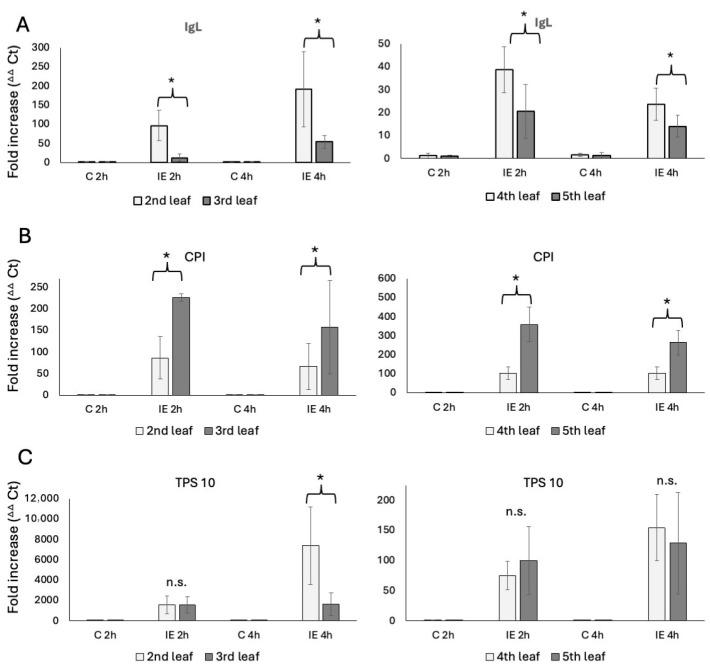
Transcript accumulation in leaves of maize seedlings in response to insect elicitor treatment (IE). N-linolenoyl-glutamine was used as an elicitor. Transcript accumulation was determined by the ^ΔΔ^Ct method [19]. (**A**) Transcript accumulation of *IgL* in V2 (left) and V4 (right) maize seedlings. (**B**) Transcript accumulation of *CPI* in V2 (left) and V4 (right) maize seedlings. (**C**) Transcript accumulation of *TPS 10* in V2 (left) and V4 (right) maize seedlings. C, control tissue; IE, tissue treated with insect elicitor. * indicates significant differences (*t* test; *p* ≤ 0.05; *N* = 3).

We used plants of the same developmental stages as described above. The transcript accumulation was analyzed from leaf samples 2 h and 4 h after IE treatment, which reflects the general maximum transcript accumulation after IE (2 h) [14,16], and the time point (4 h) when volatile emissions begin to peak [4,5]. For the V2 stage plants, transcript accumulation for *IgL* in the second leaf showed an 84 ± 36-fold increase after 2 h and a 139 ± 112-fold increase after 4 h of IE treatment (Figure 2A). The third leaf revealed a significantly lower transcript accumulation for *IgL*, with only an 11 ± 10-fold increase after 2 h and a 54 ± 16-fold increase after 4 h. No significant differences were found in control samples.

Similar results were obtained for the fourth and fifth leaves of V4 plants (Figure 2A). Here, the fourth leaf showed a 39 ± 10-fold increase after 2 h and a 24 ± 7-fold increase at 4 h after IE treatment. Again, transcript accumulation in the younger fifth leaf was significantly lower, reaching only a 21 ± 11-fold increase at 2 h and a 14 ± 5-fold increase after 4 h.

While these results correlate well with indole emissions described above, we further tested for transcripts of other defense-related genes to see if this is a general trend or specific for *IgL*. We used cysteine protease inhibitor (*CPI*) [20] and terpene synthase 10 (*TPS 10*) [21,22] as representative genes for direct and indirect defenses, respectively. For *CPI*, we found that younger leaves generally had a significantly higher transcript accumulation. In V2 plants, *CPI* accumulated 86 ± 50-fold at 2 h and 66 ± 53-fold at 4 h in the second leaf. In contrast, the younger third leaf accumulated *CPI* 226 ± 9-fold for 2 h and 157 ± 108-fold at 4 h. Similar results were found for the V4 plants, where the fourth leaf reached a 102 ± 33 fold-increase after 2 h and a 102 ± 34 fold-increase at 4 h, while the younger fifth leaf reached a 359 ± 90-fold increase at 2 h and a 263 ± 64-fold increase at the 4 h time point (Figure 2B). Clearly, the accumulation of a direct defense gene appears to be contrasting the results for *IgL* and may indicate a more targeted and direct defense activation in younger leaves of both V2 and older V4 maize seedlings.

As an indirect defense marker, we monitored *TPS 10* transcripts for terpene biosynthesis induced by insect herbivory. We found no significant differences at 2 h after IE treatment for V2 and V4 plants. In V2 plants *TPS 10* accumulated 1558 ± 869-fold in the second leaf and 1573 ± 794-fold in the third leaf, while in V4 plants *TPS 10* accumulated 75 ± 23- and 100 ± 56-fold at the same time point. However, the older second leaf of V2 plants surprisingly accumulated *TPS 10* 7386 ± 3818-fold 4 h after treatment, while the younger third leaf only reached a 1641 ± 1113-fold accumulation. In V4 plants, transcript accumulation of *TPS 10* for both leaves was not found to be significantly different at this time point (fourth leaf 155 ± 55-fold and fifth leaf 129 ± 84-fold increase) (Figure 2C).

These findings indicate that in younger leaves, protection relies much more on direct defenses and terpene emissions rather than indole.

## 3. Discussion

In recent years, indole has been established as a herbivore-induced HIPV capable of effectively priming plants against herbivores and pathogens by causing accelerated and enhanced defense responses upon subsequent damage. It has also been established that indole is often emitted earlier than other HIPVs, such as those derived from the terpene pathway, setting it apart as an earlier signaling molecule produced by plants upon insect herbivory. However, while GLVs are produced within seconds to minutes, the production of indole usually requires 2–3 h for significant quantities. Furthermore, those quantities are also lower than those of GLVs. And while GLVs are produced through a distinct pathway involving only two major enzymes, lipoxygenase and hydroperoxide lyase, indole biosynthesis is considerably more complex, primarily because indole is an intermediate of multiple pathways, each with its own distinct set of enzymes [7,8,9]. As such, indole is a precursor for benzoxazinoid defense compounds like 2,4-dihydroxy-1,4-benzoxazin-3-one (DIBOA) and 2,4-dihydroxy-7-methoxy-1,4-benzoxazin-3-one (DIMBOA) through the benzoxazinone-less (BX1-9) pathway [7,8]. Indole is also a potential intermediate of auxin biosynthesis through the tryptophane pathway. Here, indole is produced through tryptophan synthase alpha subunit (TSA) and then further processed by tryptophan synthase beta subunit (TSB1 and 2), resulting in tryptophan, which is the main precursor for auxin biosynthesis. Volatile indole as part of typical HIPVs is produced by *IgL*, which is inducible by insect elicitors during actual herbivory [11]. Indole therefore fulfills pathway-specific functions determined by the needs of the affected tissue. Interestingly, recent studies in maize demonstrated that externally applied indole is mainly converted into benzoxazinoids [23]. And while little is known about the interaction and integration of volatile indole and auxin biosynthesis, it can be expected that indole might very well be involved in the regulation of growth through auxin. The observed reduction in volatile indole in younger leaves of V2 and V4 plants might reflect a preferential allocation of the precursor, indole glycerol phosphate, towards auxin biosynthesis by reducing *IgL* expression and thus volatile indole biosynthesis. Consequently, growing leaves, as shown herein, produce reduced quantities of volatile indole but may rather keep it within the growth-regulating pathway resulting in auxin or, alternatively, channel it toward the biosynthesis of direct defense compounds such as DIBOA and DIMBOA, as shown in [23]. But while we have no evidence for either way, it would still explain why younger leaves produce significantly less indole upon treatment with an insect-derived elicitor.

A strong correlation was observed between indole emissions and *IGL* transcript levels, which are significantly reduced in younger, actively growing leaves. By contrast, the production of other HIPVs, predominately terpenes, as well as transcript levels for *TPS 10* and *CPI*, exhibited a different pattern in comparison. Combined terpenes were always emitted at significantly higher levels in younger leaves. Likewise, *CPI* appeared to be expressed much higher in younger leaves for both V2 and V4 plants, supporting the theory that younger leaves are in need of better protection. However, *TPS 10* expression varied again between V2 and V4 plants. In V2 plants, the older second leaf accumulated more transcripts for *TPS 10* than the younger third leaf despite the third leaf producing more terpene HIPVs. In V4 plants, both leaves, the fourth and the fifth, had similar levels, but as with the V2 plants, the younger fifth leaf produced more terpene HIPVs.

We can therefore conclude that transcript levels do not necessarily serve as a reliable indicator for the strength of a defense response, and other physiological factors may contribute substantially to creating a flexible response. Similarly, indole emissions as part of HIPVs are not a dependable indicator for defense intensity, as indole is negatively correlated with leaf growth in maize. As such, indole emission does not follow the general rule that younger leaves have higher degrees of defense as is shown for *CPI* expression and terpene emissions following insect herbivory. This further confirms the view that indole biosynthesis is context-dependent and as such follows distinct regulatory requirements. However, there is no doubt that indole functions as an important defense compound as it is involved in both direct and indirect defense responses. And this is where indole may have its real strength by providing plants with a flexible resource utilization and thus an effective defense strategy in a dynamical and physiologically variable environment.

## 4. Materials and Methods

### 4.1. Chemicals

Nonyl-acetate (internal standard) and indole were purchased from Sigma-Aldrich, USA. All solvents used were of analytical grade. N-linolenoyl glutamine (IE) was generously provided by Dr. Hans Alborn (USDA, ARS, CMAVE, Gainesville, FL, USA).

### 4.2. Plant Materials

Maize (*Zea mays*, var. Kandy King, J.W. Jung Seed Co., Randolf, WI, USA) plants were grown in Sungro Horticulture Professional Growing Mix (Sun Gro Horticulture Canada Ltd., Seba Beach, AB, Canada) in a growth chamber under a 12 h photoperiod at 26 °C with 60% relative humidity. The light intensity of the LED lighting was set to ca. 150 μmol m^2^ s^−1^. Growth stages were determined by following the designation outlined at https://crops.extension.iastate.edu/encyclopedia/corn-growth-stages (accessed on 3 December 2025). For all experiments, we used seedlings at the late V2 stage, with the third leaf actively growing, and at the early V4 stage, with the fifth leaf still growing.

### 4.3. Plant Treatment, Plant Volatile Collection, and Gene Transcript Accumulation

#### 4.3.1. Treatment with Insect Elicitor (IE)

For induction with IE (N-linolenoyl glutamine, 0.1 nmol/μL in H_2_O), an area of approximately 2 by 10 mm on the respective leaf of intact maize plants was scratched with a razor blade and 10 μL of this solution applied per damaged site and plant. Undamaged maize plants were used as control. We used 4 plants per treatment group. For HIPV collection, plants were treated for 3 h with IE before being cut at the mesocotyl and immediately wrapped into a wet paper towel and transferred to plexiglass cylinders for volatile collection. For gene expression analysis, maize leaves were treated with IE as described. Leaf segments from above the IE application site were used since it was shown previously that IEs only induce these genes leaf-upwards from the treatment site [17,18].

#### 4.3.2. Volatile Collection

HIPVs were collected for 2 h and were trapped on HayeSep Q80/100-containing filters (Supelco, Bellefonte, PA, USA) as described previously [4], at a flow rate of 200 mL·min^−1^ for 2 h, attached to a glass cylinder (1.8″ ID, 17″ long) containing the plant. Filters were eluted with 150 uL of dichloromethane. After adding the internal standard (Nonylacetate, 1000 ng per sample), volatile samples were analyzed on a Varian model 3900 GC coupled to Varian Saturn 2200 MS, equipped with split–splitless capillary injector systems, in electron impact mode (EI). The data collection, storage, and subsequent analysis were performed on a computer using the Varian MS Workstation software (Version 6.6). Helium at a constant flow velocity of 1 mL/min was used as a carrier gas. The analyses of the volatile collections were performed on a fused silica capillary column (Equity™ 30 m × 0.25 mm inner diameter with a 0.25 µm thick film of bonded methyl silicone). The GC was programmed as follows: initial temperature 40 °C for 2 min, then temperature programmed at 15 °C/min to 250 °C. All of the injections were made in the splitless mode. The compounds were identified by comparison to authentic standards (retention time and fragmentation).

#### 4.3.3. Transcript Accumulation (qPCR)

To test for the effects of IE on transcript accumulation of selected genes, we treated plants as described above for 2 h and 4 h. The 2nd and 3rd leaf (L2 and L3) of V2 stage plants and the 4th and 5th leaf (L4 and L5) were then cut above the IE treatment site and snap-frozen in liquid nitrogen for RNA extraction. Similar segments from undamaged plants were used as controls. Segments of leaves from three similarly treated plants were pooled for one biological replicate, and three biological replicates were performed for each treatment and time point. We extracted the total RNA from ≤100 mg of ground leaf material using the PowerPlant^®^ RNA Isolation Kit containing DNAse (MO BIO Laboratories, Inc., Carlsbad, CA, USA), with the following modifications: The frozen samples were homogenized in 2 mL screw-cap tubes containing 0.5 g of Zirmil microbeads and 200 µL of extraction buffer (PR1) for 20 s at 6000 shakes min^−1^ in a Precellys tissue homogenizer (MO BIO Laboratories, Inc., Carlsbad, CA, USA). After the initial homogenization, we added 800 µL of PR1 and homogenized the samples for an additional 10 s at 6000 shakes min^−1^. The extract was then processed according to the manufacturer’s instructions.

We used the High-Capacity cDNA Reverse Transcript Kit (Applied Biosystems, Foster City, CA, USA) to synthesize the cDNA. We performed real-time PCR using the 7300 Real-Time PCR System (Applied Biosystems). The PCR reactions were performed in a 20 µL volume containing 10 µL of SYBR Green PCR Master Mix (GLPBIO, Montclair, CA, USA), 0.2 µM of forward and reverse primer, respectively, and the cDNA equivalent to 25 ng of total RNA. The primer specificities were confirmed by melting curve analysis, and the relative transcript levels were calculated using the 2^−ΔΔCT^ method [19], with *Membrane protein PB1A10.07c* (*MEP*) as a reference gene [24]. We selected the following genes for our analysis: Indole-3-glycerol lyase (*IgL*) as the main marker for indole production [11], terpene synthase 10 (*TPS 10*) as a marker for general volatile biosynthesis [21,22], and cysteine protease inhibitor (*CPI*) as a marker for direct defenses [20].

### 4.4. Primer Sequences

*IGL: F 5′-GTCTATCTCGCGAGCGTCAA, R 5′-TGATCACACCATCTGCACCC*
*TPS10: F 5′-TGTGTCCACGGTCCAATGTT, R 5′-GTCCGCTGTCCTTGCAAAA*
*CPI: F 5′-GGACATGAGCTGGCGATTTT, R 5′-CAAGGAGCACAACAGGCAGA*
*MEP: F 5′-TGTACTCGGCAATGCTCTTG, R 5′-TTTGATGCTCCAGGCTTACC*

### 4.5. Statistical Analysis

Data are presented as means ± standard deviation (SD) of at least three independent biological replicates for each treatment and time point. Data were analyzed for significance in a pairwise comparison with Student’s *t*-test (*p* ≤ 0.05) (Microsoft Excel, Version 16.88).

## 5. Conclusions

Indole emissions in response to simulated insect herbivory are significantly regulated by the developmental status of the leaf rather than by the leaf’s need for effective defense.

This regulation likely reflects indole’s broader role within the overall physiology of the plant, as it participates in multiple metabolic pathways, and places indole in a distinctive position among defense compounds.

## Data Availability

The raw data supporting the conclusions of this article will be made available by the authors on request.

## Abbreviations

The following abbreviations are used in this manuscript:

GLVs	Green leaf volatiles
TPS 10	Terpene synthase 10
CPI	Cysteine protease inhibitor
DIBOA	2,4-dihydroxy-1,4-benzoxazin-3-one
DIMBOA	2,4-dihydroxy-7-methoxy-1,4-benzoxazin-3-one
IAA	Indole acetic acid
MAPK	Mitogen-activated protein kinase
HIPVs	Herbivore-induced plant volatiles
DMNT	4,8-dimethyl-1,3,7-nonatriene
IE	Insect elicitor
IgL	Indole glycerol phosphate lyase
TSA	Tryptophan synthase alpha subunit
TSB	Tryptophan synthase beta subunit
BX	Benzoxazinone-less

## References

[1] Kessler A., Baldwin I.T. (2002). Plant responses to insect herbivory: The emerging molecular analysis. Annu. Rev. Plant Biol..

[2] Engelberth J., Baluska F., Witzany G. (2012). Plant resistance to insect herbivory. Biocommunication in Plants.

[3] Mauch-Mani B., Baccelli I., Luna E., Flors V. (2017). Defense Priming: An Adaptive Part of Induced Resistance. Annu. Rev. Plant Biol..

[4] Engelberth J., Alborn H.T., Schmelz E.A., Tumlinson J.H. (2004). Airborne signals prime plants against insect herbivore attack. Proc. Natl. Acad. Sci. USA.

[5] Erb M., Veyrat N., Robert C.A., Xu H., Frey M., Ton J., Turlings T.C. (2015). Indole is an essential herbivore-induced volatile priming signal in maize. Nat. Commun..

[6] Matsui K. (2006). Green leaf volatiles: Hydroperoxide lyase pathway of oxylipin metabolism. Curr. Opin. Plant Biol..

[7] Richter A., Powell A.F., Mirzaei M., Wang L.J., Movahed N., Miller J.K., Piñeros M.A., Jander G. (2021). Indole-3-glycerolphosphate synthase, a branchpoint for the biosynthesis of tryptophan, indole, and benzoxazinoids in maize. Plant J..

[8] Florean M., Luck K., Hong B., Nakamura Y., O’Connor S.E., Köllner T.G. (2023). Reinventing metabolic pathways: Independent evolution of benzoxazinoids in flowering plants. Proc. Natl. Acad. Sci. USA.

[9] Florean M., Schultz H., Grabe V., Luck K., Kunert G., O’Connor S.E., Köllner T.G. (2025). A pseudoenzyme enables indole biosynthesis in eudicot plants. Nat. Chem. Biol..

[10] Sun P., Huang Y., Yang X., Liao A., Wu J. (2023). The role of indole derivative in the growth of plants: A review. Front. Plant Sci..

[11] Frey M., Stettner C., Pare P.W., Schmelz E.A., Tumlinson J.H., Gierl A. (2000). An herbivore elicitor activates the gene for indole emission in maize. Proc. Natl. Acad. Sci. USA.

[12] Ye M., Liu M., Erb M., Glauser G., Zhang J., Li X., Sun X. (2021). Indole primes defense signaling and increases herbivore resistance in tea plants. Plant Cell Environ..

[13] Ye M., Glauser G., Lou Y., Erb M., Hu L. (2019). Molecular Dissection of Early Defense Signaling Underlying Volatile-Mediated Defense Regulation and Herbivore Resistance in Rice. Plant Cell.

[14] Engelberth J., Contreras C.F., Dalvi C., Li T., Engelberth M. (2013). Early transcriptome analyses of Z-3-Hexenol-treated *Zea mays* revealed distinct transcriptional networks and anti-herbivore defense potential of green leaf volatiles. PLoS ONE.

[15] Yoshinaga N., Ishikawa C., Seidl-Adams I., Bosak E., Aboshi T., Tumlinson J.H., Mori N. (2014). N-(18-hydroxylinolenoyl)-L-glutamine: A newly discovered analog of volicitin in Manduca sexta and its elicitor activity in plants. J. Chem. Ecol..

[16] Wang L., Jaeggi S., Cofer T.M., Waterman J.M., Walthert M., Glauser G., Erb M. (2023). Immature leaves are the dominant volatile-sensing organs of maize3. Curr. Biol..

[17] Engelberth J., Contreras C.F., Viswanathan S. (2012). Transcriptional analysis of distant signaling induced by insect elicitors and mechanical wounding in Zea mays. PLoS ONE.

[18] Köllner T.G., Lenk C., Schnee C., Köpke S., Lindemann P., Gershenzon J., Degenhardt J. (2013). Localization of sesquiterpene formation and emission in maize leaves after herbivore damage. BMC Plant Biol..

[19] Livak K.J., Schmittgen T.D. (2001). Analysis of relative gene expression data using real-time quantitative PCR and the 2(-Delta Delta C(T)) Method. Methods.

[20] Tanaka Y., Fujita K., Date M., Watanabe B., Matsui K. (2023). Structure-activity relationship of volatile compounds that induce defense-related genes in maize seedlings. Plant Sig Behav..

[21] Köllner T.G., Gershenzon J., Degenhardt J. (2009). Molecular and biochemical evolution of maize terpene synthase 10, an enzyme of indirect defense. Phytochemistry.

[22] Seidl-Adams I., Richter A., Boomer K.B., Yoshinaga N., Degenhardt J., Tumlinson J.H. (2015). Emission of herbivore elicitor-induced sesquiterpenes is regulated by stomatal aperture in maize (*Zea mays*) seedlings. Plant Cell Environ..

[23] Sorg A., Luo Z.W., Li Q.B., Roy K., Basset G.J., Kim J., Hunter C., Chapple C., Rering C., Block A.K. (2025). The airborne herbivore-induced plant volatile indole is converted to benzoxazinoid defense compounds in maize plants. New Phytol..

[24] Manoli A., Sturaro A., Trevisan S., Quaggiotti S., Nonis A. (2012). Evaluation of candidate reference genes for qPCR in maize. J. Plant Physiol..

